# To stay or re-migrate after the pandemic shock? Labor re-migration intention to the coastal areas of Thừa Thiên Huế province in Vietnam

**DOI:** 10.1016/j.heliyon.2023.e18765

**Published:** 2023-07-27

**Authors:** Le Thi Hoa Sen, Jennifer Bond, Tien Dung Nguyen, Thi Hong Mai Nguyen, Dung Ha Hoang, Chung Nguyen Van, Tran Thi Anh Nguyet, Quang Phuc Nguyen

**Affiliations:** a Hue University of Agriculture and Forestry, Hue University, 102 Phung Hung Str., Hue City, Viet Nam; b Gulbali Institute for Agriculture, Environment and Water, Charles Sturt University, P.O Box 789, Albury, NSW, 2640, Australia; c University of Economics, Hue University, 99 Ho Dac Di str. Hue City, Viet Nam

**Keywords:** Remigration, Behavioral intention, Determinants, Coastal area, Extended theory of planned behavior

## Abstract

This study applied the extended theory of planned behavior (ETPB) to explore factors that shape the behavioral intention of migrant laborers returning to the coastal region of Thừa Thiên Huế province during the peak of the COVID-19 pandemic to remigrate. Data collection included 210 interviews with returned migrants aged 18 and above in 4 coastal communes of Thừa Thiên Huế province, in-depth interviews (8 key informants) and a group discussion (5 persons). Exploratory factor analysis and a multivariate linear regression model were applied to analyze the data. Results showed that in addition to individual socio-demographic characteristics (i.e. level of education, age, income level and sex), attitudes, subjective norms, perceived behavioral control, and perceived risk were also determinant factors of migrants’ intention to remigrate. This research suggested that in order to build resilience of rural migration workers, related departments should seriously consider the following recommendations: (i) raise awareness and encourage young rural people to complete their education and necessary skills; (ii) organize related psychological training programs for rural laborers; (iii) raise awareness about the necessity to participate in social insurance; (iv) promote rural job creation programs suitable for low education and low-skilled rural laborers; (v) raise awareness for local people, particularly school children, about the value and opportunities of being smart farmers in the context of 4.0 technology and social risks at the destination for low education and low-skilled laborers; (vi) digitalize migration labor management and provide digital information services regarding job opportunities for rural laborers.

## 1 Introduction

Migration is among the most debated issues in rural development of developing countries and Vietnam is no exception. Within Vietnam, the *Doi Moi* policies of the 1980s opened up the country to decollectivized agriculture as well as population mobility [1,2]. Internal migration has increased sharply over recent decades in Vietnam [3], where approximately 13% of the population are considered internal migrants [4]. The proportion of female migrants has also increased, and approximately 50% of internal migration is rural-urban or rural-rural migration and most migrants are born in rural areas [4]. The majority of migrants participate in ‘spontaneous migration’ (i.e. migration that has occurred outside of a government program) [3] that has resulted in socioeconomic development, social security and resilience challenges for both rural people and those at the destination [5,6]. A large number of previous studies investigated drivers of out-migration of rural people, finding that drivers were diverse and depended considerably on the socio-economic context [3]. However, the migration flow tends to be from poorer or vulnerable areas to areas that are wealthier and less vulnerable [6,7]. For example, in Vietnam, people migrate from rural areas of the North or North Central to the southern or central highlands; from mountainous areas to big cities such as Hồ Chí Minh, Hà Nội, or Đà Nẵng [4].

Drivers for internal migration have been studied for decades and diversified fundamental dimensions of drivers have been identified, including social, institutional/political, economic, cultural, demographic, and ecological factors [8,9]. These drivers are often termed as ‘push’ and ‘pull’ factors based on whether they are associated with the place of origin or destination [10]. The number of divers and levels of influence of each driver is context-specific. Economic drivers are often the most significant, followed by social factors, such as education and medical access [10]. For the economic factor, better earnings or income opportunities are key to motivate people to migrate, leaving their family in order to stay in the urban areas. Many migrants aim to earn money, which they can save and send home periodically for their family [8,9,11].

Poor people employ their assets through a number of income strategies to ensure livelihoods for their family [12]. Migration may be a strategy to deal not only with shocks but also to diversify livelihoods [13] and reduce poverty. Rural-urban migration can have impacts on both the original place through movement of labour out of the agricultural sector, as well as impacts at the destination through pressure on infrastructure and services such as housing [13]. Indeed, there are instances of migration leading to only children and old people left at the place of origin, supported by remittances [14]. Livelihood resources, particularly agricultural land resources, are underutilized in the place of origin. Alternatively, those rural migrants who move to urban areas, don't necessarily reach their livelihood or reduced poverty goals [15].

Migrants have been identified as among the most vulnerable group to impacts of the COVID-19 pandemic [16]. Many migrant laborers were left unemployed, without income and were vulnerable to socio-psychological risk during the lockdown period of the COVID-19 pandemic [[17], [18], [19]]. In Vietnam, mass waves of migrants (thousands of migrants), returned home during the peak period of COVID-19 to their rural areas in the central provinces [14]. The movement of migrants to the rural areas caused challenges for the local government and rural communities in ensuring food security, social and psychological wellbeing, disease control and livelihood sustainability in the long-term. Therefore, migrants, particularly those working in unorganized sectors are a socio-economically vulnerable group and potentially a risk to communities at the destination as well as at the origin. This precarious position begs the question: *will these labourers re-migrate and if so, what are the drivers of this remigration*?

Drivers of out-migration have been investigated widely within the literature, but drivers of remigration after returning to the place of origin after a shock, have been given less attention. Many studies related to decision-making in a challenging or unstable environment are related to organizational decisions, rather than the individual [20,21]. These studies showed that making a decision in or after a shock is faster and less comprehensive than in a stable environment without stressful conditions. Under pressure or stressful conditions, the organization's leaders have to make quick decisions to ensure the continuous functioning of the organization's business [22]. However, for the individual, the decision-making may take longer since individuals have to take into account more issues of risk and uncertainty with limited capacity and information. This can lead to a prolonged decision-making processes and even waiting to see others' success and following them [23].

Coastal areas of Thừa Thiên Huế have diversified livelihood activities, including crop cultivation, livestock keeping, aquaculture production, offshore fishing, aquatic resource processing (i.e. fish sauce), working as waged labor and out-migration. However, these communities’ livelihoods are increasingly vulnerable to multiple stressors such as climate change, exhausted aquatic resources, marine environmental shocks and unstable small-scale aquaculture production [24]. With this background, the region is famous for both domestic and international out-migration. The national or rural-urban migration has been increasing over time, mainly for young workers, with the peak wave of rural-urban migration in the region after the marine environmental pollution shock in 2016 [24,25]. This wave of movement was considered as a livelihood adaptation strategy for the majority of small-scale households most affected [25]. Many of these households have kept their land resources and other livelihood capital at their homeland for security when facing risk at the migration destination, while others sold all their livelihood resources, such as land, house, fishponds or fishing facilities before migrating [26]. This may lead to different levels of livelihood vulnerability of the workers who have now returned due to COVID-19.

This paper aims to investigate the factors shaping migrant workers’ intention to re-migrate after returning to their place of origin due to COVID-19 impacts. We focus on those migrants who returned to the coastal areas in the central provinces of Vietnam, where the majority of workers, particularly young workers, moved to southern Vietnam in 2016.^1^ Large numbers of these migrants returned to the central provinces during the peak period of the COVID-19 pandemic in Vietnam. Therefore, we aim to fill a gap in the literature regarding the behavioral intention of returned migrants to remigrate.

## 2 Methodology

### 2.1 Theoretical framework

**Fig. 1:**
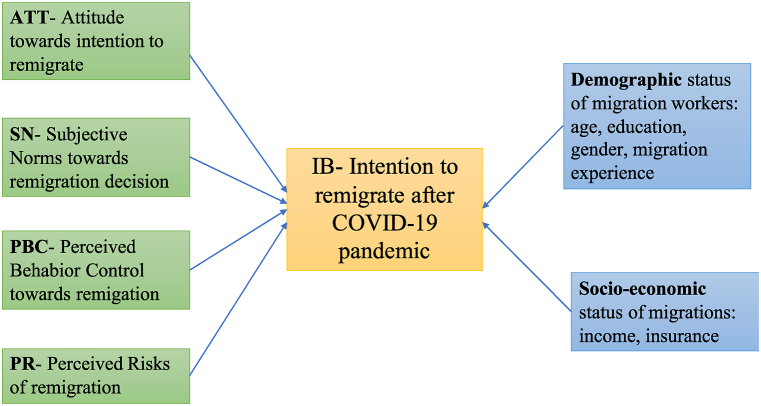
Theoretical frame work of intention of migrants to remigrate after the COVID-19 pendamic

The Theory of Planned Behavior (TPB) is a well-developed and tested framework through which to analyze behavior [27] and it has been widely used to analyze the factors influencing farmers' intention to adopt a livelihood strategy [28,29]. After experiencing a social shock due to the COVID-19 pandemic and in the context of rapid social, economic and environmental changes, people's livelihoods, particularly migrants, face several challenges. Migrants may have increased concern about social and economic risks when they decide to remigrate [17,18,30]. It is, thus, important to consider risk perception and integrate risk perception in the TPB to analyze intention of returned migrants to remigrate. Therefore, an extended TPB which comprises the original TBP [31], social factors, and risk theory [32] was employed to analyze return-migrants’ intention to remigrate after the COVID-19 pandemic (Fig. 1).

The Indicators under each latent variable were derived from previous studies and from in-depth interviews with key-informants in the studied areas.

***Subjective Norm*** (SN) is one of the important latent variables of the TPB model developed by Ajzen [31]. According to Ajzen, subjective norm refers to social pressure that makes a person perform a particular behavior. In this study, subject norm represents the pressure from family members, neighbors, community, or friends towards a migrant's intention to re-migrate. Social pressure commonly positively influences the decision to migrate [[33], [34], [35]] and thus is assumed to positively influence intention to re-migrate. This study focuses on the individual level subjective perceptions of norms [36].

Perceived behavioral control (PBC) is the second core latent variable indicating the perceptions of an individual towards the feasibility or own confidence and capacity to take the action. Thus, individual returned migrants’ intention to re-migrate would be high when they perceive that they are knowledgeable, capable, and skillful in ensuring income activities and suitable living arrangements at the destination in the context of post COVID-19 pandemic.

***Attitude (ATT)***: is the third key latent variable of the model defined by Ajzen [31] indicating “the degree to which an individual favorably or unfavorably assesses the behavior being examined.” Thus, individuals' attitudes affect their desire to re-migrate. Farmers’ attitude towards re-migration can be related to the perceived importance of re-migration to their livelihoods. When they perceive positive consequences and are interested in re-migration, they are more willing to pay for a better livelihood activity [37,38].

***Perceived risk (PR):*** Perceived risk is an important variable in adoption behavior analysis [39]. Perceived risk is defined as a kind of subjective loss or possible loss when people try to pursue a desired result [40]. Quite a large number of studies have examined the impact of perceived risk on decision-making, particularly in adoption of digital services or a new technology [41]. But to our knowledge there have been no studies integrating PR in TPB to examine migrants’ intention to re-migrate. This re-migration is in the context of post-COVID-19 pandemic, where people may face significant livelihood risks and uncertainty. As there was substantial socio-economic and security uncertainty at the peak period of the pandemic, re-migration is thus perceived of as high risk in terms of social security and joblessness [42].

### 2.1.1 Social indicators

Previous adoption studies show that individuals' socio-economic characteristics such as age, education level, gender and annual income level are important for making decisions [[43], [44], [45]]. Therefore, these factors were expected to have a significant effect on the intention and decision to re-migrate. A person's status of having social insurance in Vietnam is an indicator of job and income stability [46] so it may influence migrants' intention to re-migrate. Out-migration experience (years of out-migration) is likely to also be an important indicator for decision to re-migrate according to some key informants. This study, thus, tried to combine these social indicators with variables of the Extended TPB (ETPB) to analyze migrants' behavioral intention.

### 2.2 Site selection

**Fig. 2:**
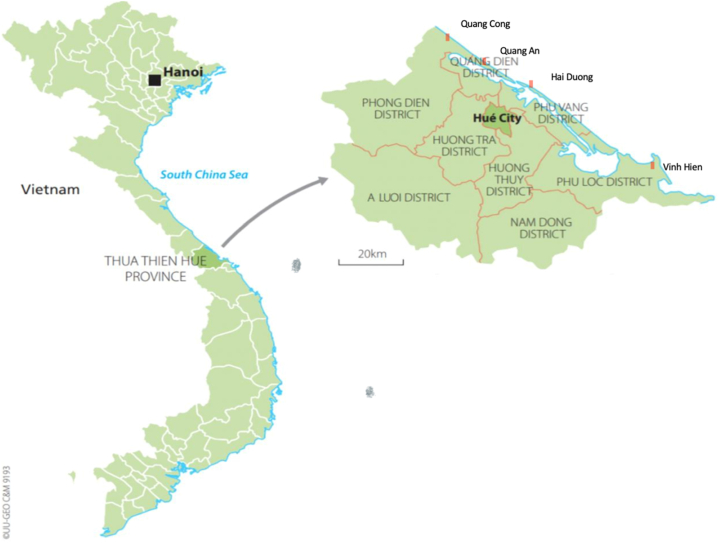
Location of the studied communes.

Source: Modified from Haase et al. [49]

The coastal area of Thừa Thiên Huế (TTH) stretches along 120 km of coastline. People have diversified livelihood activities, including crop cultivation, livestock keeping, aquaculture production, offshore fishing, aquatic resource processing (i.e., fish sauce), working as waged laborers, and out-migration. However, due to poor natural resources (limited land areas with major characteristic of white-sand dunes and poor fertility) and geographical conditions (location), the coastal area of Thừa Thiên Huế province has been considered as a significantly vulnerable area to multiple stressors, including climate change, environmental pollution and natural resource exploitation [25]. These factors contribute to TTH becoming a well-known area for out-migration in Vietnam. In addition, out-migration has been seen as an adaptive livelihood strategy of the central coastal communities [26]. The central coast is among the regions that received the highest number of returned migrant workers during the COVID-19 pandemic [14,47,48]. Quảng Công, Quảng An, Hải Dương and Vinh Hiền are among 19 coastal communes along the TTH coastline. Based on the provincial Department of Labor, Invalids and Social Affairs (DOLISA) and Department of Communication and Information, these communes had the highest number of returning migrant laborers during the COVID-19 pandemic in the coastal areas of TTH province. Further, these communes were labelled as “green” during the pandemic, indicating no movement restrictions applied during our field survey period. Therefore, we were able to access these communities for data collection. The location of the four studied communes is shown in Fig. 2.

### 2.3 Sample size and data collection

Data for the study was collected from household surveys, in-depth interviews of commune and village leaders and a group discussion. Households were selected randomly from the list of households having migrant labor (age 18 and above) registered with related departments after they had returned home during the peak period of the COVID-19 pandemic. Since Explanatory Factor analysis (EFA) was employed in this study, an appropriate sample size needed to be determined. According to Hair et al. [50], the minimum sample size for this study was 105 (21 observed variables/items x 5). The actual sample size of this study was 210, which satisfied the sample size requirement. A closed-questionnaire covering 21 indicators of ETPB was designed and undertaken face-to-face with returned migrant workers. The questionnaire also included respondents’ demographic characteristics, job, income and livelihood assets. The questionnaire was pre-tested prior to the main survey and adjusted accordingly.

The ETPB indicators were identified based on reviews of literature, eight in-depth interviews of key informants (commune and village leaders) and one group discussion. The group discussion was conducted with the participation of commune leaders, commune Women's Union and an expert working at provincial DOLISA. The group discussion focused on verifying ETPB indicators for this research. We proposed to invite 11 people: 2 from each studied commune and 3 from provincial DOLISA. However, due to the COVID-19 situation, some could not participate. Only five people participated in the group discussion, however some studies suggest that smaller-sized groups, such as with five people, allow for greater dialogue [51]. The transcripts of the interviews and group discussion were analyzed through content analysis using a deductive approach guided by the original TPB framework. Questions broadly explored the status of out-migration of local workers, their return during COVID-19 and socio-economic dimensions of these movements. The content analysis allowed for the ETPB model appropriate for this study to be developed.

The study methods and protocol were approved by the Research Ethics Committee for Social Sciences and Humanities of Hue University (No.2000/QĐ-ĐHH dated December 16, 2021). All methods were carried out in accordance with relevant guidelines and regulations. An informed consent form was obtained from all participants for data collection.

### 2.4 Data analysis

Reliability measure of the scale was performed, together with an EFA and multiple regression analysis. Since all ETPB indicators are measured by a Likert scale, Cronbach's alpha test was used to test the reliability of the assessment items and the principal component analysis (PCA) extraction method and Varimax rotation were used for factor analysis. All assessment items needed to have a Cronbach's alpha coefficient greater than 0.7, otherwise they were removed from the adjusted index [52]. As a result, 4 items/observed variables were removed from the ETPB indexes: 01 of the ATT; 01 of SN and 02 of the PBC. Each latent variable (ATT, SN, PBC and PR) as well as the behavioral intention variable (BI) was scored through the mean of its indicators. Multiple regression was applied to analyze factors shaping migrants' intention to re-migrate after returning home due to the COVID-19 pandemic. The dependent variable was the mean score of intention indicators (BI) and independent variables were latent variables of ETPB and migrants' social variables.

## 3 Results

### 3.1 Socio-demographic information of respondents

Table 1 presents major socio-demographic information of interviewed migrant workers. Most respondents were young laborers with the mean age of 33.7 years old. The value of SD is 10.8, showing a relative variation of labor age, with some laborers older than 60 in the sample. There were more females than males in the sample (51.9% female and 48.1% male). Most respondents had a high education level. Nearly 42% of respondents completed secondary school and 49% completed high school and university. Only 9.4% had finished their education at primary school. On average, respondents had 7.9 years of out-migration experience. Around 96% of respondents had health insurance but only 17% had social insurance. Their average annual income was 39.3 million VND/year (equal to ∼$1,700 USD). This income was after deducting meal/food costs (and accommodation for some respondents) at work paid by employers.

**Table 1:** Socio- Demographic information of interviewed migrant labors (N = 210).

Description characters	Unit	Mean	SD
Age of migration labour	Year	33.7	10.8
Gender			
Male	%	48.1	–
Female	%	51.9	–
Education level of migration labour	Class	9.95	2.4
Illiteracy	%	0	
Primary school	%	9.4	
Secondary school	%	41.6	
High school and upper	%	49.0	
No of year out-migration	Year	7.9	5.9
Having health insurance	%	96.1	–
Having social insurance	%	17.0	–
Average annual income level	Million VND/year	39.3	28.7

**Fig. 3:**
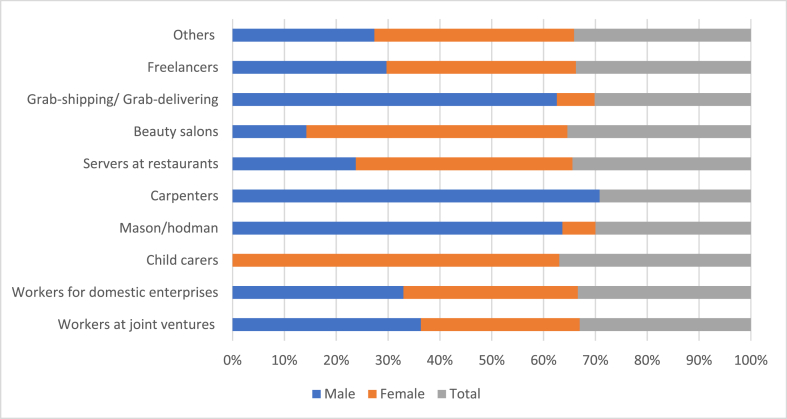
Jobs at the destination of returned migrants. Note: Male (n = 101), Female (n = 109).

Fig. 3 presents diversified job types of the interviewed migrant laborers at their destination. The majority of respondents worked for domestic enterprises/companies (34.56%), such as agricultural processing enterprises, ceramic tile companies and paper manufacturing companies; freelancers (33.23%); and for joint ventures, such as mobile phone, shoes, and fabric companies (14.77%). The ratio of male and female laborers is equally distributed in these job types. This indicates males and females equally access these jobs at the destination. Some jobs were mainly for males, such as carpentry, masonry, Grab shipping or Grab delivering. Conversely, other jobs were mainly for females, including childcare and work in beauty salons. Most of these jobs did not require a high level of education but did require skills. However, respondents did comment on the improved opportunities for those with higher education, with one respondent stating:

*the availability of alternative jobs in urban areas (destination) has been getting more competitive. Overtime, with new technology, they require less labor force but higher qualifications. However, it is also a chance for many highly qualified laborers to get better payment. This is the biggest challenge for the majority of the rural labors but also the incentive for many of us to concentrate on improving capacity*.

### 3.2 Exploratory factor analysis (EFA)

Before conducting EFA, a scale reliability test was performed. The test results of latent variables ATT, SN, PBC, PR and BI met the requirements with Cronbach's alpha coefficient >0.5 (Table 2) and the corrected item-total correlation coefficient of each indicator (sub variable) as well as for the entire set of indicators was >0.3 (appendix 1 and appendix 2). The EFA analysis was conducted for both independent and dependent variables. The KMO index was 0.791 and Bartlett's was significant at < 0.0001 (Table 3 and Table 5), which meet the conditions for applying the EFA model. The total variance explained was 73.4% for 4 factors (Table 4). Table 4 also shows that attitude contributes highest to the variation of the independent variables (36.707%), followed by PBC (17.995%) and then subjective norms (10.317%).

**Table 2:** Results of reliability test of the scale.

Latent variables	Items (indicators)		Cronbach's alpha
	Before	After	
Attitude	5	4: ATT1, ATT2, ATT4, PBC3	0.717
Subjective norm	4	3: SN1, SN2, SN4	0.810
Perceived behavior control	6	4: PBC1, PBC2, PBC4, PBC5	0.819
Perceived risk	3	3: PR1, PR2, PR3	0.747
Behavioral Intention	3	3: BI1, BI2, BI3	0.654

**Table 3:** KMO and Bartlett's test of independent variables.

Kaiser- Meyer- Olkin Measure of Sampling Adequacy	Bartlett's test of Sephericity		
	χ2	df.	Sig.
0.791	1559.629	91	0.000

**Table 4:** Results of exploratory factor analysis of ETPB- independent variables.

Indicators		Factor loading	Eigenvalue	Variance explained (%)	Cumulative variance explained (%)
**Attitude (ATT)**			**5.139**	**36.707**	**36.707**
ATT4	There are more job opportunities that are suitable to my capacity outside of my home village	0.896			
ATT3	I can achieve a better income so that I can ensure my family's livelihood outside my home village	0.848			
ATT1	I am able to save more money when I stay away from my family and my home village	0.845			
PBC3	There are more policies on welfare for migration laborers	0.806			
**Subjective norm (SN)**			**1.444**	**10.317**	**65.019**
SN1	I can't find a suitable job at my home village while my financial resources have almost run out	0.800			
SN2	There is no other way than being a farmer. (Local norm: the best life is escaping from farming)	0.795			
SN4	Local people consider me as jobless and dependent on my family	0.711			
**Perceived Behavior Control (PBC)**			**2.519**	**17.995**	**54.702**
PBC1	I am confident that I can manage my life well at the destination after COVID-19 (stronger after a shock)	0.759			
PBC2	With my skills and knowledge, I am confident to find a good job after COVID-19	0.768			
PBC5	Safety and security related policies will be promoted by authorities at the destination after COVID-19	0.723			
PBC4	I have enough financial resources to get health and social insurance and to have a safe life at the destination after COVID-19	0.802			
**Risk Perception (RP)**			**1.119**	**7.992**	**73.011**
PR1	Overall, remigration is a social risk because I have to leave my family and my home village	0.791			
PR2	Remigration is a risk for my livelihood because of the labor market uncertainty post pandemic	0.709			
PR3	Remigration is a risk for me and my family because I have lost my job and all other assets and need to start from beginning.	0.754

**Table 5:** EFA test for dependent variable.

Indicators		Factor loading	Eigenvalue	Cronbach's alpha	Variance explained (%)	Cumulative variance explained (%)
**Behavioural Intention (BI)**						
BI1	I intend to remigrate when COVID-19 is under control	0.868	1.961	0.714	65.358	65.358
BI2	I will remigrate and find ways for an adaptive life at the destination	0.790	0.641	0.514	21.363	86.721
BI3	I will encourage my friends and others to remigrate	0.763	0.398	0.672	13.279	100.00
	Kaiser- Meyer- Olkin Measure of Sampling Adequacy	0.644				
	Bartlett's test of Sphericity:	χ2: 143.346				
		df: 3				
		Sig. 0.000				
	Cumulative % Eigenvalues					

### 3.3 Multiple regression results

The regression analysis was performed to identify factors shaping migrant laborers intention to re-migrate after the COVID-19 pandemic. Results of the multiple regression showed that the F test has a value of 45.01 at P < 0.001 indicating the multiple regression model is suitable. The maximum variance inflation factor coefficient (VIF) was 1.477 < 2, meaning that no collinearity existed in the model. The R^2^ = 0.462, indicating that the model is acceptable [53] and the variation of all independent variables can explain 46.2% of the variation of the dependent variable.

Table 6 shows that attitude, and education level significantly positively influenced migrant laborers’ intention to re-migrate (P < 0.001). This result implies the need for special attention to these two factors to support rural migrant laborers. Further, perceived behavioral control, perceived risk and years of out-migration significantly influenced behavioral intention (P < 0.05), annual income and gender (sex) also influence their intention to re-migrate (P < 0.01). Some respondents felt confident about their financial status and ability to pay for setting themselves up and performing a new job in the destination if they re-migrate. A respondent in Hải Dương commune expressed that:

*I think that there would be a lot of challenges ahead if we re-migrate but I believe that with my experience and capacity it is not too difficult to find a good-paid job at the destination. We have to be confident to face challenges, then we may get good opportunity. If we are hesitant and self-doubt, and just keep waiting and following others, it is hard to get good chances*.

**Table 6:** Regression results: factors determining decision to re-migrate after COVID-19 pandemic.

Variables	Un-standardized Coefficient	Std. Error	Standardized Coefficients	t.value
Attitude	0.323	0.046	0.398***	6.890
Perceived behavior control	0.161	0.052	0.195**	3.084
Subjective norm	0.091	0.049	0.113*	1.866
Perceived Risk	−0.118	0.040	−0.161**	−2.944
Annual income (million VND)	0.005	0.003	0.098*	1.842
Education level of migrants (grade 1-12)	0.048	0.013	0.206***	3.778
Having social insurance (0-no insurance)	−0.001	0.072	−0.001	−0.021
Age of migrants	−0.001	0.001	−0.027	−0.468
Gender (0-male) Years of out migration	−0.122 0.013	0.057 0.004	−0.113* 0.163**	−2.133 3.074
Constant	1.334***	0.295		4.477
R^2^	0.462			

Note: ***, ** and * are significant levels at 1%, 5% and 10% level of probability, respectively.

Perceived Risk and gender negatively influenced the dependent variable, indicating that the more risks migrants perceive, the less they intend to re-migrate. In addition, females were less likely to intend to re-migrate than males. Comparing the regression coefficient among all independent variables, attitude has the highest value (0.398), followed by education level (0.206) and perceived behavioral control (0.195), meaning that attitude was the most important factor influencing re-migration intention, followed by education level and perceived behavioral control. Average annual income, gender (sex) and subjective norm had the lowest coefficient value with 0.098; 0.113; and 0.113, respectively, meaning these dimensions were less influential to intention than the other factors. Social insurance and age of migrants were shown to have no significant influence on migrants’ intention to re-migrate. However, the older the migrants are, the less interest they have for re-migration. Some experienced respondents considered the pandemic as a shock and also a chance for them to return to their village for a safe life. A 51-year-old migrant expressed:

*I have migrated for almost 26 years. I migrated with a group of four friends. I have tried to work with different jobs with the aim of getting as much income as possible and send home for children’s education and for having a concrete house at home. I have spent most of my life with a very hard-working environment. In the past years I am wondering if I should return back to my village and if I can earn a good living with a similar job here but I couldn’t decide because my friends and my family members didn’t support it. Experiencing all the socio- political problems that happened during the COVID-19 pandemic in the southern provinces, particularly at my workplace, I recognized my unsafe and insecure life. It was the most terrible shock in my life. I decided to return to my village permanently. I already had a good house, my children still need support but with my skills and experience now I believe I can manage household expenses at the rural area. I feel happy and safe when staying with my family. It’s the best for the rest of my life.*

## 4 Discussion

The application of ETPB with the regression analysis method found that the two most important factors that influence respondents’ behavioral intention include education level and attitude. The higher level of education, the more likely that migrants intend to remigrate after COVID-19 pandemic. This is logical, as highly educated workers often try to get well-paid jobs in urban, rather than rural areas. The better educated workers often have greater opportunities to secure improved employment or are prioritized above people in the urban areas with lower education levels. In addition, most rural areas in Vietnam are still poor. Rural livelihoods rely on agriculture, particularly the poor households of the study areas. There is, thus, limited jobs available for highly-educated migrants. This finding confirms results of previous research [24,25,54].

Regression results also showed that attitude was a critical variable determining intention to re-migrate. This implies that job opportunities, income level and opportunities to strengthen their capacity are the major influences on respondents' intention to re-migrate. The urban areas often offer more job opportunities and more chances to choose jobs suiting migrants' capacity than the rural areas. Further, higher income levels in urban areas were crucial for migrant laborers to ensure the livelihoods of their family at home through remittances. Due to the limited resources and multiple livelihood risks in rural areas, respondents were unable to find many opportunities to ensure their livelihoods to support their family, and therefore, out-migration was seen as the best choice to ensure continuous income and remittances for their family. These findings confirmed results of previous research [55] that found that job opportunities and income level are the most critical drivers for internal migration. This study shows that economic drivers remain a core factor in re-migration intention after a shock. Further, capacity building was also an important attribute of migrants’ attitude towards intention to re-migrate, a factor found through the application of the ETPB and therefore, has not been directly reported by previous studies.

The results also showed that subjective norm is a critical factor for re-migration intention. This implies that individuals' perceptions of social norms influenced respondents' intention to re-migrate. It was not pressure from family members to re-migrate but from neighbors and from traditional customs and norms. One of the typical traditional customs of rural Vietnamese society, particularly in the central coastal areas, is the discussion on private issues of others. In some respects, this indicates social capital, collective action and communities' support for each other to build resilience. However, in other respects, this creates social problems. Respondents expressed that they may be considered jobless and dependent on their family if they don't re-migrate. Other respondents worried about their community thinking they have no prospects other than being a farmer or can't escape from farming. These ways of thinking and communication within neighborhoods considerably influenced respondents' re-migration intention. However, one key informant suggested that this was more of a perception held by returned migrants rather than a widely held community norm.

Community perceptions have also been changing. Nowadays, with development of technologies in agriculture and the availability of agricultural land (due to a high percentage of farmers leaving agriculture to out-migrate or looking for non-farming activities) many farmers have become skillful and wealthy with improved livelihood capitals. Their quality of life is perceived to be better than that of families with migrants. Therefore, local people have started changing their perception of leaving farming. In addition, with the development of digital agriculture together with the social security and safety challenges faced by migrant laborers at the destination during the COVID-19 pandemic, the local customs and norms about escaping from farming may change faster. This study found that some returned migrants, particularly old or capable migrants intend to not re-migrate. This finding was not consistent with results of recent studies [56,57], which showed that there has been some “reverse migration from urban to rural” in some rural areas of Vietnam, but this has been restricted to very few, capable laborers who were able to capitalize on 4.0 technology and underutilized agricultural resources in their rural areas as a result of big waves of out-migration in the past decade.

Risk perception was also a significant factor of re-migration intention. Due to the shock of the pandemic, respondents are becoming more cognizant of socio-economic risks of re-migration. Respondents were concerned with various risks, such as social security when living away from family. They also worry about the risks of unemployment and financial loss (for traveling and living cost at the destination) if they re-migrate due to the uncertainty of the labor market post- COVID-19. Although respondents were concerned by multiple risks, behavioral control was also found to be among three critical factors for re-migration intention. This implies that in relation to intention to re-migrate, confidence is vital. Respondents need to feel confident about their financial status and ability to pay for setting themselves up and performing a new job at the destination if they re-migrate. Each of these significant factors of TPB have as yet not been seen in previous migration research.

This is perhaps a key demarcation between more general drivers of migration and migration associated with a shock. General migration literature has outlined that ‘push’ and ‘pull’ factors drive migration, essentially in relation to the place of origin or destination [10], noting that these are context specific. In relation to shocks, studies have often focused on migrants' return to their place of origin, such as after the Asian Financial Crisis [58], or out-migration as a coping strategy such as after an environmental shock (i.e. [59,60]). Studies focusing on migrants' return to the place of origin due to a shock and then subsequent re-migration are few. COIVD-19 provides an example of investigating this kind of re-migration. Other studies focusing on COVID-19 migration in low-income countries highlight the livelihood vulnerabilities of returning migrants [14], which could be hypothesized as a driver for post-COVID re-migration as many rural households are dependent on remittances, particularly after a shock [61]. However, studies from China show that migration home at the onset of the pandemic and re-outmigration after the pandemic can be difficult, where some migrants were discriminated against based on ethnicity, through continued restrictions and transport policies [62]. Our respondents did not perceive difficulty in returning home at the start of the pandemic due to the Vietnamese authorities' decision that all provinces allow returning migrants through a “No one left behind” message promoted through social media. While our study's findings that attitude towards job opportunities and education level are the most influential on behavioral intention to re-migrate post-COVID-19, further research should be done in the Vietnamese context to understand any difficulties these re-migrants may have returning to cities.

Besides factors from ETPB, this research also found that years of out-migration, gender and average annual income also influence respondents’ intention to remigrate. The more years of being migrants, the more interest respondent had to return to the destination for work and living in the urban area. However, the older respondents have less interest to leave family and the rural area. Male laborers were more likely to intend to re-migrate than females. This may be due to male laborers in the areas being more responsible for the family livelihood/income than females. They, thus, were under greater pressure to re-migrate to access better paid opportunities in the urban areas. This finding is consistent with data reported by others [5,63] regarding migration and gender issues.

Income level has been found to be the most critical variable for migration decision in many previous studies [8,9,11]. It was also found to be important for re-migration intention in this study, however, it was not the key factor. Its regression coefficient was relatively small (0.098) implying that the level of income (high or low) was not really a major concern for respondents’ intention to re-migrate. It can be explained that not only laborers with high level of income intend to re-migrate, some low-income respondents also had strong intentions to re-migrate. It was reviewed by some respondents that it is better to have a small income and small remittances than no income if staying at the home village. They mean that having something is better than nothing.

Results of the regression also showed that years of out-migration and social insurance did not significantly influence re-migration intention. This suggests that not only experienced migrants but also new migrants intended to re-migrate and not only those with social insurance but also those without social insurance have a similar mood to re-migrate. However, most respondents expressed that after the pandemic shock they pay more attention to social insurance. They recognized the necessity of having insurance in a changing society. It may help reduce negative consequences of unemployment and other socio-economic shocks. This finding confirmed the research results of Linh et al. [64] regarding social protection for rural-urban migrants in Vietnam.

### 4.1 Study limitations

While the data collection was able to be carried out relatively freely, there were still some COVID restrictions that impinged on the activities undertaken, such as limited access to key informants for our group discussions. However, the scoping nature of the purpose of the group discussions, for development of the subsequent survey instrument rather than for decision-making, reduced the impact of this limitation on the study.

By the nature of drawing on an ETPB, our research has focused on the behavioral intentions to migrate of the individual who was interviewed, and we have not focused on other household members' migration which some studies have found to be significant to migration intention [58]. Similarly, as we have focused on the individuals’ perceptions of social norms, and further work could focus on incorporating community level perceptions of social norms around re-migration in the post-COVID era. In addition, this paper employed the PCA extraction method and Varimax rotation for factor analysis but not principal axis factoring (PAF). This might be also a limitation of this study.

## 5 Conclusion and implications

Results of this research showed that after returning to their home village due to the COVID-19 shock, migrants are making decisions more carefully, taking into account more security and safety issues. Results also confirmed that using the Extended Theory of Planned Behavior could be a suitable framework for exploring factors shaping intention to re-migrate after a shock. It showed that attitude, subjective norm, perceived behavioral control and risk perception were associated with behavioral intention as proposed by the ETPB. This research confirmed that besides individual socio-demographic characteristics, including level of education, age, income level and gender (sex), attitude, subjective norm, perceived behavioral control and risk perception were also determinant factors of migrants’ intention to re-migrate. These findings are crucial for policy makers and related stakeholders to build resilience of rural migrant workers. Related departments and organizations, particularly the provincial DOLISA, Department of Agriculture and Rural Development (DARD), Department of Continuous Training and Education and community-based organizations working with migrant laborers should consider these findings in order to improve their strategic plans related to rural migration. These departments should seriously consider the following recommendations: (i) raise awareness and encourage young rural people to complete their education and necessary skills development; (ii) organize training programs regarding overcoming shocks and managing life during shocks) for rural laborers, particularly for those who intend to migrate; (iii) raise awareness about the necessity to participate in social insurance; (iv) promote rural-job creation programs suitable for low education and low-skilled rural laborers; (v) raise awareness of local people, particularly school children about the value and opportunities of being dynamic farmers in the context of 4.0 technology, and the social risks at the destination for low educated and low-skilled laborers; (vi) digitalize migration labor management and provide digital information services on job opportunities for rural laborers. The digital migration management system, including migration registration, purpose, intended destination and personal information on capacity and skills may help reduce risks through additional training courses as required.

## Funding

This work is funded by the Vietnam National Foundation for Science and Technology.

Development (NAFOSTED) under grant number: 04.2021/ÐX.

## Author contribution statement

Thi Hoa Sen Le, PhD: Conceived and designed the experiments; Performed the experiments; Contributed reagents, materials, analysis tools or data; Wrote the paper.

Jennifer Bond: Conceived and designed the experiments; Contributed reagents, materials, analysis tools or data; Wrote the paper.

Dung Tien Nguyen: Conceived and designed the experiments; Performed the experiments; Analyzed and interpreted the data.

Mai Thi Hong Nguyen; Ha Dung Hoang; Nguyet Thi Anh Tran: Performed the experiments.

Chung Van Nguyen; Phuc Quang Nguyen: Performed the experiments; Analyzed and interpreted the data.

## Data availability statement

The authors do not have permission to share data.

## Declaration of competing interest

The authors declare that they have no known competing financial interests or personal relationships that could have appeared to influence the work reported in this paper.

## Footnotes

1 The Formosa Environmental Disaster 2016 in Vietnam.
