# Supplementary material for: To stay or re-migrate after the pandemic shock? Labor re-migration intention to the coastal areas of Thừa Thiên Huế province in Vietnam
Source: Heliyon. 2023 Jul 27;9(8):e18765. doi: 10.1016/j.heliyon.2023.e18765 (PMC10404773; doi:10.1016/j.heliyon.2023.e18765)
Supplement: Multimedia component 1 [file mmc1.docx]

Appendix 1: Cronbach’s Alpha and corrected item-total correlation test

| Indicators | Cronbach’s alpha | Corrected item-total correlation test | Cronbach's Alpha if Item Deleted |
| --- | --- | --- | --- |
| **Subjective norm** | **0.810** |  |  |
| SN1 |  | 0.651 | 0.756 |
| SN2 |  | 0.717 | 0.677 |
| SN4 |  | 0.651 | 0.771 |
| **Attitude** | **0.888** |  |  |
| ATT3 |  | 0.748 | 0.858 |
| ATT4 |  | 0.845 | 0.819 |
| ATT1 |  | 0.720 | 0.868 |
| PBC3 |  | 0.706 | 0.874 |
| **Perceived Behaviour Control** | **0.819** |  |  |
| PBC1 |  | 0.622 | 0.786 |
| PBC2 |  | 0.662 | 0.766 |
| PBC4 |  | 0.709 | 0.742 |
| PBC5 |  | 0.587 | 0.798 |
| **Perceived Risks** | **0.747** |  |  |
| R1 |  | 0.600 | 0.633 |
| R2 |  | 0.601 | 0.637 |
| R3 |  | 0.529 | 0.722 |
| **Behavioural Intention** | **0.729** |  |  |
| B1 |  | 0.493 | 0.714 |
| B2 |  | 0.651 | 0.514 |
| B3 |  | 0.538 | 0.672 |

Appendix 2: EFA for entire set of TBP indicators

| Latent variables | Indicators | Corrected Item-Total Correlation | Cronbach's Alpha if Item Deleted |
| --- | --- | --- | --- |
| ATT- Attitude | ATT1 | 0.432 | 0.857 |
|  | ATT2 | 0.506 | 0.853 |
|  | ATT3 | 0.509 | 0.853 |
|  | ATT4 | 0.570 | 0.849 |
| PBC- Perceived Behavioral Control | PBC1 | 0.496 | 0.853 |
|  | PBC2 | 0.544 | 0.852 |
|  | PBC3 | 0.563 | 0.850 |
|  | PBC4 | 0.481 | 0.854 |
| SN- Subjective Norm | SN1 | 0.497 | 0.853 |
|  | SN2 | 0.595 | 0.847 |
|  | SN3 | 0.584 | 0.850 |
| PR- Perceived Risks | R1 | 0.508 | 0.853 |
|  | R2 | 0.504 | 0.853 |
|  | R3 | 0.462 | 0.856 |
